# Accurate 3D LiDAR SLAM System Based on Hash Multi-Scale Map and Bidirectional Matching Algorithm

**DOI:** 10.3390/s24124011

**Published:** 2024-06-20

**Authors:** Tingchen Ma, Lingxin Kong, Yongsheng Ou, Sheng Xu

**Affiliations:** 1 Guangdong Provincial Key Laboratory of Robotics and Intelligent System, Shenzhen Institute of Advanced Technology, Chinese Academy of Sciences, Shenzhen 518055, China; tc.ma@siat.ac.cn (T.M.); lx.kong@siat.ac.cn (L.K.); 2 University of Chinese Academy of Sciences, Beijing 100049, China; 3 Department of Electronic and Electrical Engineering, Southern University of Science and Technology, Shenzhen 518055, China; 4 Faculty of Electronic Information and Electrical Engineering, Dalian University of Technology, Dalian 116024, China

**Keywords:** hash multi-scale map, bidirectional matching, LiDAR SLAM, robot navigation

## Abstract

Simultaneous localization and mapping (SLAM) is a hot research area that is widely required in many robotics applications. In SLAM technology, it is essential to explore an accurate and efficient map model to represent the environment and develop the corresponding data association methods needed to achieve reliable matching from measurements to maps. These two key elements impact the working stability of the SLAM system, especially in complex scenarios. However, previous literature has not fully addressed the problems of efficient mapping and accurate data association. In this article, we propose a novel hash multi-scale (H-MS) map to ensure query efficiency with accurate modeling. In the proposed map, the inserted map point will simultaneously participate in modeling voxels of different scales in a voxel group, enabling the map to represent objects of different scales in the environment effectively. Meanwhile, the root node of the voxel group is saved to a hash table for efficient access. Secondly, considering the one-to-many (1 $\times \;10^{3}$ order of magnitude) high computational data association problem caused by maintaining multi-scale voxel landmarks simultaneously in the H-MS map, we further propose a bidirectional matching algorithm (MSBM). This algorithm utilizes forward–reverse–forward projection to balance the efficiency and accuracy problem. The proposed H-MS map and MSBM algorithm are integrated into a completed LiDAR SLAM (HMS-SLAM) system. Finally, we validated the proposed map model, matching algorithm, and integrated system on the public KITTI dataset. The experimental results show that, compared with the ikd tree map, the H-MS map model has higher insertion and deletion efficiency, both having O(1) time complexity. The computational efficiency and accuracy of the MSBM algorithm are better than that of the small-scale priority matching algorithm, and the computing speed of the MSBM achieves 49 ms/time under a single CPU thread. In addition, the HMS-SLAM system built in this article has also reached excellent performance in terms of mapping accuracy and memory usage.

## 1. Introduction

Compared with visual SLAM [1,2,3], LiDAR SLAM is widely used in the industrial field due to its robustness in complex scenes (lighting changes and low textures). Currently, most LiDAR SLAM [4,5,6,7] systems use direct point cloud maps with a “point” being the smallest unit for environmental modeling. During the localization process, each measurement point will be associated with a landmark represented by a specific number of neighboring map point sets. However, because the point set with a specified number may not achieve optimal landmark modeling, it will further limit the accuracy of state estimation and subsequent map updating. On the other hand, by maintaining voxels of different scales [8,9], objects of various sizes in the environment can be accurately represented. However, compared with point cloud maps, maintaining voxel maps requires the preset voxel segmentation threshold or the estimation of the scene size, which increases the difficulty of using the map. Therefore, it is important to design a practical mapping method that is convenient to use and has a high map modeling accuracy for diverse, complex scenes.

For point-based map [10] models, researching universal scene representations and improving maintenance efficiency are currently hot topics. The authors of [11,12] proposed a structured feature extraction method based on scan smoothness. Then, the accumulated multi-frame feature sets were used to construct a structured point cloud map. An interesting map modeling method was proposed in [13], in which an implicit moving least squares surface composed of local points was developed. Compared with [11,12,14], the method of [13] is also suitable for unstructured scenes. Cai et al. [15] proposed a modified ikd tree model that supports increment point insertion and deletion. Consequently, compared with the traditional kd tree model, the new model does not need to rebuild a new tree structure when inserting new map points. The ikd tree map has been widely applied in different open-source SLAM systems [16,17].

For voxel-based [18] map models, the current research hotspot is to improve the map model accuracy. Biber et al. [19] considered constructing the environment as a normal distribution model within the fixed-size voxel, which can effectively represent complex scenes and achieve reliable positioning and mapping. Yuan et al. [8] used a specific threshold to segment voxels in the hash octree map. Thus, each voxel in the map contained a unique plane landmark. Undoubtedly, the accuracy and robustness of mapping depend on the threshold selection. In [20], Nguyen et al. used the improved octree model [9] to represent the environment. The plane landmarks of different scales are simultaneously maintained in corresponding voxels. However, this method requires manually presetting the maximum depth of the octree map by estimating the size of the scene. Unlike the above studies, this paper proposes a hash multi-scale map model that does not require presetting the voxel segmentation threshold or adjusting the maximum depth parameter according to the scene size.

On the other hand, to achieve robust and accurate robot localization, the SLAM system requires some corresponding matching algorithms. At present, the matching method based on “point” has been thoroughly studied. For example, some improved iterative closest point (ICP) [21,22] algorithms used the covariance distribution of measurement and target points during registration. Therefore, these algorithms can work well in complex scenarios. In [23,24], different methods were proposed to extract edge or surface feature points from point clouds, and then state estimation was achieved by matching feature points to the local feature map. In addition, the method of [25] introduced the intensity information of point clouds during the registration process, which further improves registration accuracy.

Recently, some research has focused on registering point clouds to voxel landmarks. Biber et al. [19] proposed a method matching each 3D measurement point with 27 neighboring voxels via the normal distribution model. Due to its ability to achieve one-to-many matching, this algorithm had strong robustness but slightly low accuracy. Liu et al. [26] developed a kd tree with the mean of all points in the voxel. Then, using the nearest matching solution, each feature point was associated with a unique voxel landmark by the kd tree. For the matching problem of multi-scale models, Nguyen et al. [20] proposed a small-scale priority matching algorithm to achieve the data association between the measurement and modified octree map. However, the premise of this algorithm is that small-scale map voxels can always achieve higher accuracy in modeling. This condition does not always hold in practice. On the other hand, in the multi-scale map model, the number of small-scale landmarks is usually much larger than that of larger scales. Therefore, traversing and comparing the smaller-scale landmarks will be a time-consuming process. To address the issues above, we propose a novel multi-scale bidirectional matching algorithm (MSBM). The core idea of this algorithm is to construct a candidate set using only a limited number of neighboring voxels at the to-be-searched scale.

The main contributions of this article are concluded below.

- A novel hash multi-scale (H-MS) map model is proposed. This model can be used without estimating the scene size or presetting the voxel segmentation threshold.
- A multi-scale bidirectional matching (MSBM) algorithm, adapted to the H-MS map model, is developed. The algorithm can achieve satisfactory performance in the trade-off problem between registration accuracy and efficiency.
- The proposed map model and matching algorithm are integrated into a unified SLAM (HMS-SLAM) system. The proposed algorithm and system are compared with some state-of-the-art methods based on public datasets.

The remaining part of the article is organized as follows. Section 2 presents the proposed map model and matching algorithm. Section 3 introduces the integrated SLAM system. Section 4 and Section 5 detail the experiments and conclusions, respectively.

## 2. Method

### 2.1. Problem Formulation

In this article, we present a novel hash multi-scale (H-MS) map model designed to achieve precise scene modeling by concurrently maintaining voxel landmarks at different scales. Additionally, we employ a hash table to store multi-scale voxel groups, ensuring efficient data queries.

The H-MS map is maintained through point insertion and voxel deletion (single voxel, frame-based) operations. The temporal complexity of point insertion and single-voxel deletion operations is O(1) . Unlike existing methods such as the hash adaptive voxel map [8], which relies on predetermined thresholds for voxel segmentation, and the octree map [20], which requires estimation of scene size to specify maximum depth parameters, our model overcomes these limitations.

In addition, the proposed H-MS map model has a low matching efficiency because of the high computational complexity. To address this problem, we introduce a multi-scale bidirectional matching (MSBM) algorithm to enhance the efficiency and robustness of data association.

### 2.2. Hash Multi-Scale Map

This subsection introduces the proposed hash multi-scale map model from three aspects: data structure, map maintenance (insertion, deletion), and data querying.

#### 2.2.1. Data Structure

In the H-MS map, each key value in the hash table connects to a root voxel node (the map voxel with the largest scale $s_{0}$ (unit=m) ) of the multi-scale voxel group. By defining the maximum depth *d* of the multi-scale map, the scale of voxels in other layers can be calculated by

(1) sn=s0s02n2n

where *n* indicates that the voxel is located at the *n*-th (n=0,1,…,d−1) scale of the group. The voxel coordinate $v_{0}$ at scale $s_{0}$ can be derived by forward projecting the 3D point p to the map coordinate system *m*. Utilizing the hash function, we calculate the mapping relationship between $v_{0}$ and the key value key in the hash table, thus obtaining the corresponding root node ${Ø}_{0}$ in the H-MS map, and we have

(2) p=[px,py,pz]Tv0=pps0s0=[v0,x,v0,y,v0,z]Tkey=hash_combine(v0)

where hash_combine() is the function used in the open-source boost library to generate unique key values for the hash table. Based on the specified scale $s_{n}$ , the voxel coordinate $v_{n}$ corresponding to p can be obtained through the forward projection operation, i.e.,

(3) vn=ppsnsn=[vn,x,vn,y,vn,z]T.

**Figure 1:**
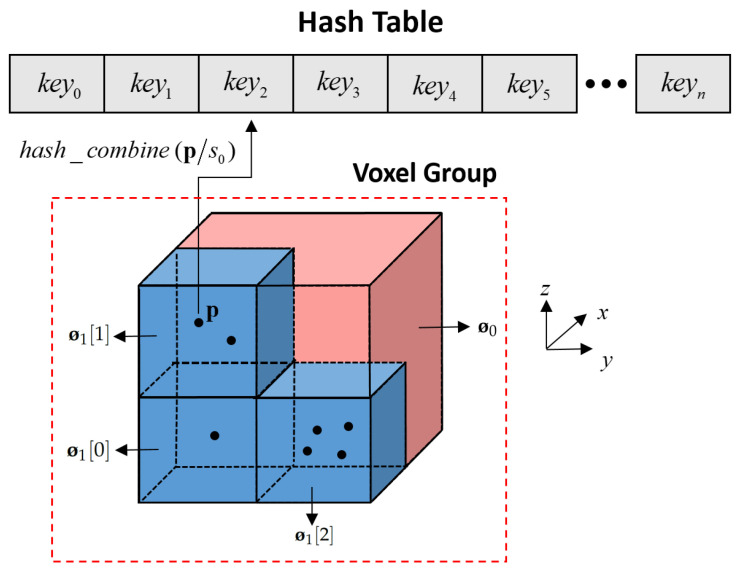
An example of the H-MS map. ${Ø}_{0}$ is a map voxel at scale $s_{0}$ , ${Ø}_{1}\left[0\right]$ , ${Ø}_{1}\left[1\right]$ , and ${Ø}_{1}\left[2\right]$ are map voxels at scale $s_{1}$ . Map point p participates in the calculation of statistical features for both ${Ø}_{0}$ and ${Ø}_{1}\left[1\right]$ simultaneously.

Because a map point will participate in the creation of voxel landmarks across all scales within the group, increasing the maximum depth *d* enhances map modeling accuracy. Furthermore, each voxel in the H-MS map maintains statistical features: point number *N*, mean u , and covariance ∑. The plane, edge, or normal distributed landmarks can be calculated from the statistical features. To manage memory usage, a frame-based voxel deletion method is also implemented in the H-MS map. Consequently, each map voxel needs to maintain an observation count variable $l^{obs}$ . Figure 1 illustrates an example of the H-MS map.

#### 2.2.2. Map Maintenance

The insertion and deletion of the H-MS map are operated in units of “point” and “voxel”, respectively. The reason for voxel deletion rather than point deletion is that the H-MS map aims to construct a multi-scale map based on voxel landmarks. Therefore, deletion is always performed with the voxel as the smallest unit.

**Point insertion:** For a given scale $s_{n}$ , assuming that the voxel coordinate of p at that scale is $\left(v_{n,x},v_{n,y},v_{n,z}\right)$ , the following equation determines the insertion position $leaf_{id}$ of the map voxel at the next scale:

(4) $$\left\{\begin{array}{c} q=\left[\begin{array}{c} p_{x}<\left(s_{n}v_{n,x}\right)?0:1\\ p_{y}<\left(s_{n}v_{n,y}\right)?0:1\\ p_{z}<\left(s_{n}v_{n,z}\right)?0:1 \end{array}\right]\\ leaf_{id}=4*q\left[0\right]+2*q\left[1\right]+1*q\left[2\right]. \end{array}\right.$$

The process of inserting a map point can be divided into two steps: (1) calculating the corresponding root node pointer ${Ø}_{0}$ in the hash table, and (2) recursively inserting points. The pseudocode is illustrated in Algorithm 1.
**Algorithm 1:** Map point insertion
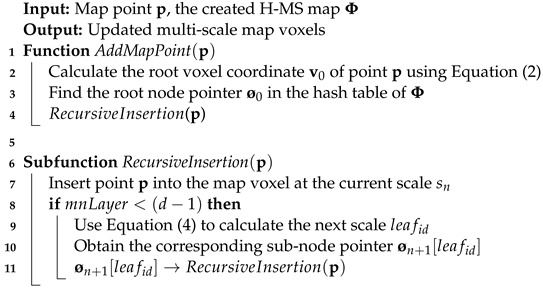


**Voxel deletion:** The H-MS map supports both single-voxel and frame-based voxel removal methods. Firstly, we use a method for removing a single voxel. Given the scale $s_{n}$ , we define the map coordinate m as the center position of the voxel to be deleted, and the corresponding processing method is introduced below.

(1) Perform a forward projection of m with scale $s_{0}$ to obtain the root voxel coordinate $v_{0}$ by
(5) vo=mms0s0.
(2) Utilize Equation (4) recursively to find and delete the voxel node at scale $s_{n}$ .

The frame-based voxel deletion method can be defined if the map is organized by frame. Firstly, when a frame of point cloud enters the map, the “point insertion” operation is used to update the H-MS map. All observable map voxels are stored in vector form, with the observation count $l^{obs}$ adding 1.

Then, when removing an inserted frame from the map, operations happen in the stored voxel vector. By traversing the voxel vector, the observation count $l^{obs}$ stored in the voxel will subtract 1. We can observe whether the observation count $l_{0}^{obs}$ of each root voxel is zero to determine whether the voxel group can be removed. The reason why we use the observation count $l_{0}^{obs}$ to delete the voxel group is that a map point is simultaneously inserted into voxels of different scales. The observation count $l_{0}^{obs}$ of the root voxel is always equal to or greater than those of smaller-scale voxels.

#### 2.2.3. Data Query

The H-MS map supports querying *k* neighboring voxels at a specified scale, where *k* can take values of 1, 7, or 27. Firstly, the reverse projection operation is defined as projecting the voxel coordinate $v_{n}$ into the map coordinate system *m* through scale $s_{n}$ to calculate the map coordinate m , which takes the form

(6) $$m=s_{n}v_{n}.$$

Taking map point p as an example, the query process for 27 neighboring voxels in scale $s_{n}$ can be outlined as:

(1) Calculate the nearest voxel coordinate vnc of p through the forward projection of Equation (3). Then, the set of neighboring voxel coordinates $V_{n}=\left\{v_{n}^{1},\ldots ,v_{n}^{i},\ldots ,v_{n}^{27}\right\}$ can be represented as stacking $\left[-1,0,1\right]$ on each dimension of coordinate vnc .
(2) Utilize Equation (6) and (5) to perform reverse and forward projection operations on the voxel coordinate set $V_{n}$ . This yields the corresponding map coordinate set $M=\{m^{1},\ldots ,m^{i},\ldots ,m^{27}\}$ and root node voxel coordinate set $V_{0}=\left\{v_{0}^{1},\ldots ,v_{0}^{i},\ldots ,v_{0}^{27}\right\}$ .
(3) Recursively apply Equation (4) to the map coordinate set M to obtain the required voxel set at scale $s_{n}$ .

### 2.3. Multi-Scale Bidirectional Matching Algorithm

The multi-scale bidirectional matching algorithm is designed based on the H-MS map to associate measurement point clouds with corresponding voxel landmarks. During the data association process, each measurement point undergoes three operations: forward, reverse, and forward projections to obtain the candidate voxel set. The best match pair will be generated from the candidate voxel set. Subsequently, the algorithm is developed considering the matching target, evaluation criteria, and matching method.

#### 2.3.1. Matching Target and Evaluation Criteria

Considering the generality of surface features in real scenes, we use the plane parameter ßm=[nm,dm]T calculated from the accumulated measurements in each voxel as the landmark (refer to Section 3.1.3 for the calculation method). Assuming that the point set in the LiDAR coordinate system *l* to be registered is Ω=p1l,…,pil,…,pnl , the algorithm’s objective is to obtain the matching set U=p1l→ßbest1,…,pil→ßbesti,…,pnl→ßbestn .

Assuming that the predicted pose that can transform the measurement point pil to the map coordinate system *m*, ${\hat{T}}_{ml}\in SE\left(3\right)$ . The corresponding rotation matrix and translation vector are ${\hat{R}}_{ml}\in SO\left(3\right)$ and ${\hat{t}}_{ml}\in R^{3}$ , respectively. The distance from the point pil to the candidate plane landmark ßm can be defined as

(7) eiß=ßTmT^mlp˜il

where p˜il is the homogeneous coordinate of pil . We use the absolute value $\left|e^{ß}\right|$ as the evaluation criteria for matching.

#### 2.3.2. Matching Method

Taking the measurement point p˜il as an example, Algorithm 2 provides a specific method for obtaining the matching relationship between the point p˜il and the plane landmark ${ß}_{j,best}^{k}$ .

In Algorithm 2, line 1 calculates the predicted 3D point in the map coordinate system *m*. Lines 2–3 initialize the scale vector. Lines 4–13 calculate the candidate landmark set at multiple scales and sort them based on evaluation criteria. Lines 14–18 perform a distance check on the obtained matching pairs with minimum criteria.

In addition, it can be seen that the candidate matching set for each measurement point is composed of a specified number of neighboring voxel sets at different scales. For instance, with the maximum depth *d* of the H-MS map being set to 3 and considering the neighboring set with 27 voxels, there can be up to 81 candidate-matching landmarks in the priority_queue . Compared with the small-scale priority matching algorithm [20], the proposed method enables an equitable search for the best landmark across all scales and utilizes a smaller candidate set.
**Algorithm 2:** Multi-scale bidirectional matching algorithm
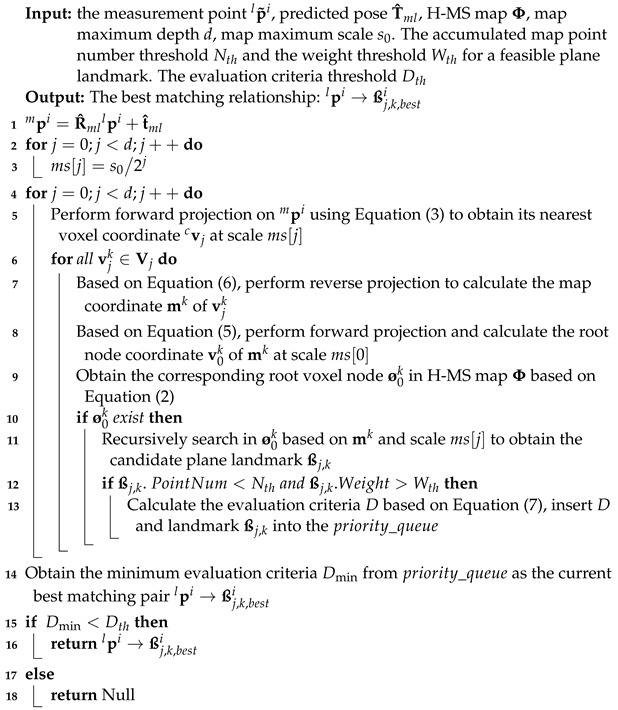


## 3. System

**Figure 2:**
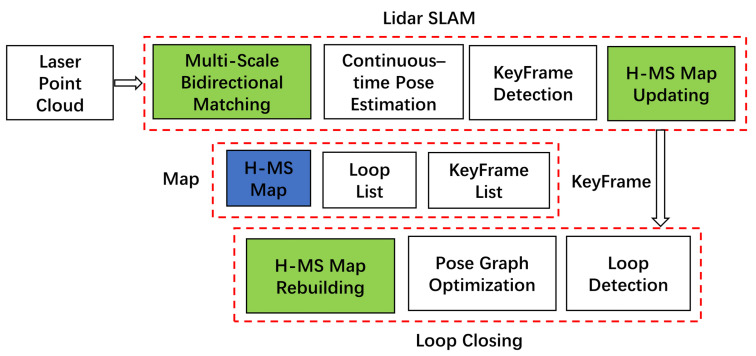
The HMS-SLAM system flowchart.

This section introduces the 3D LiDAR SLAM framework that integrates the proposed H-MS map and MSBM algorithm. Figure 2 depicts the system flowchart. The localization and mapping module discussed in Section 3.1 and the loop closure module outlined in Section 3.2 are executed in two separate threads.

### 3.1. Localization and Mapping

#### 3.1.1. Robot Localization Based on the MSBM Algorithm

To enhance the computational efficiency of localization and mapping, we employ the method proposed in [13] to process the raw point cloud data and obtain a sampling point set Ω=p1l,…,pil,…,pnl . Then, Algorithm 2 is utilized to achieve data association U between the sampling point set Ω and H-MS map Φ .

Because the robot is typically mobile during the continuous sampling process of the LiDAR sensor, it is necessary to process motion distortion when estimating the robot pose using a raw point cloud [23]. Assume that the initial and final poses of the current frame $F_{j}$ are $X_{j}=\left[T_{j}^{s},T_{j}^{e}\right]\in \mathrm{SE}{\left(3\right)}^{2}$ , with corresponding timestamps $\left[\eta_{j}^{s},\eta_{j}^{e}\right]$ . The localization module can utilize the data association U and the following equation to construct a cost function for estimating $X_{j}$ , i.e.,

(8) $$\min_{X_{j}}\sum_{i=1}^{n}{∥{\bar{e}}_{i,j}^{ß}\left(X_{j}\right)∥}_{2}^{2}+\theta_{t}{∥t_{j}^{s}-t_{j-1}^{e}∥}_{2}^{2}$$

where the second term represents the inter-frame localization consistency constraint. $\theta_{t}$ is the corresponding weight coefficient, which is proposed in [27]. ${\bar{e}}_{i,j}^{ß}\left(X_{j}\right)$ denotes a point-to-plane error considering distortion correction,

(9) e¯i,jß=nbestipi,jm+dbesti

where point pi,jm in the map coordinate system *m* is obtained by transforming the measurement point pi,jl with

(10) pi,jm=Rjλipi,jl+tiλiRjλi=slerpqjs,qje,λitjλi=1−λitjs+λitje.

In Equation (10), $\left[q_{j}^{s},q_{j}^{e}\right]\in \mathrm{SO}{\left(3\right)}^{2}$ represents the rotational component of $X_{j}$ , and $\left[t_{j}^{s},t_{j}^{e}\right]\in {\left(R^{3}\right)}^{2}$ represents the translation component of $X_{j}$ . The slerp() function calculates the spherical linear interpolation between two quaternions. $\lambda^{i}$ denotes the relative timestamp, which has

(11) λi=ηi−ηsηi−ηsηe−ηsηe−ηs.

Finally, we employ the LM algorithm [28] to solve Equation (8).

#### 3.1.2. Keyframe Detection

When both the time interval and relative motion transformation conditions are satisfied, the system will create a new keyframe:

(1) Time interval: Assume that the time when the last keyframe was inserted is $\eta_{last}^{kf}$ . If the interval between $\eta_{last}^{kf}$ and $\eta_{j}^{e}$ (the end timestamp of current frame $F_{j})$ exceeds the threshold $\eta_{th}^{kf}$ , condition (1) holds.
(2) Relative motion transformation: Calculate the relative transformation $T_{rel}^{kf}$ based on the end pose $T_{j}^{e}$ of the current frame $F_{j}$ and the pose $T_{last}^{kf}$ of the last inserted keyframe. If the relative translation part $t_{rel}^{kf}$ exceeds threshold $t_{th}$ or the relative rotation part $R_{rel}^{kf}$ represented by Euler angle exceeds threshold $R_{th}$ , condition (2) holds.

When a keyframe needs to be created, the system utilizes the estimation result $X_{j}$ from Section 3.1.1 to correct the motion distortion of the sampling point set Ω . Then, the end pose $T_{j}^{e}$ of the current frame $F_{j}$ and distortion point cloud $\bar{\Omega }$ are stored in the new keyframe $F_{j}$ . Finally, $F_{j}$ will be inserted into the sliding window S and keyframe list H .

#### 3.1.3. H-MS Map Updating

The system utilizes keyframes in the sliding window S to maintain a local H-MS map, where each voxel stores the independent statistical feature $\Lambda^{k}=\left[N^{k},u^{k},\sum^{k}\right]$ and the plane parameter ßkm=[nkm,dkm]T . For the newly inserted keyframe $F^{cur}$ , we employ its pose $T^{cur}$ to transform $\bar{\Omega }$ to the map coordinate system *m*. Then, Algorithm 1 recursively inserts each map point into the H-MS map. All map voxels observed by the point cloud in $F^{cur}$ are stored as a vector.

To accelerate the H-MS map updating step, the system first updates the statistical features of the smallest-scale map voxels using a “point increment” method. Assuming that the statistical features of the current map voxel are the point number ${N^{k}}^{\prime }$ , mean ${u^{k}}^{\prime }$ , and covariance ${\sum^{k}}^{\prime }$ , then the new statistical features updated by pNkm (keyframe index *j* is omitted) can be represented as:

(12) Nk=Nk′+1uk=(Nk′uk′+pNkm)/Nk∑k=1Nk′∑i=1Nk(pim−uk)(pim−uk)T=1Nk′∑i=1Nk(pim−uk′+uk′−uk)(pim−uk′+uk′−uk)T=1Nk′Nk′∑k′+(pNkm−uk′)(pNkm−uk′)T+(Nk+1)(uk′−uk)(uk′−uk)T+2(uk′−uk)(pNkm−uk′)T.

The statistical feature at other scales can be recursively obtained from smaller scales using the method proposed by [20]. Assume that the eigenvalues of covariance $\sum^{k}$ in descending order are $\left[\alpha_{0},\alpha_{1},\alpha_{2}\right]$ . The plane parameters in each voxel can be updated. The normal vector nkm is the feature vector corresponding to $\alpha_{0}$ , and the offset is dkm=−nkm·uk . The weight of the plane landmark is calculated by W=(α1−α2)(α1−α2)α0α0 .

When the number of keyframes in the sliding window S exceeds $K_{sl}$ , the system will perform a “minus 1” operation on the observation count $l_{0}^{obs}$ of all visible voxels in the earliest keyframe. When the observation count $l_{0}^{obs}$ of the root voxel returns to zero, the voxel group will be removed from the hash table. Finally, the earliest keyframe is deleted from the sliding window S .

### 3.2. Loop Detection and Correction

#### 3.2.1. Loop Detection

When a new keyframe $F^{cur}$ enters, the system constructs a kd tree based on the translation component of each keyframe in the list H . Then, the translation component $t^{cur}$ of the current keyframe is searched within the kd tree to obtain a set of neighboring historical keyframes. If the difference between the timestamp of a historical keyframe $F^{his}$ and the timestamp of the current keyframe exceeds $\eta_{th}^{loop}$ , the system will create a local point cloud map. The map comprises $n^{loop}$ historical keyframes before and after $F^{his}$ .

By using the ICP [29] algorithm, the measurement point cloud stored in the current keyframe $F^{cur}$ can be registered to the local map. The registration error is defined as $e_{loop}$ and the correction transformation is $T_{correct}^{cur}$ . When $e_{loop}$ is small enough, it means that a loop can be found. Assuming the estimated pose of the historical keyframe $F^{his}$ is $T^{his}$ , then the relative motion transformation $T_{h,c}^{loop}$ from $F^{cur}$ to $F^{his}$ can be computed by

(13) $$T_{h,c}^{loop}={T^{his}}^{-1}T_{correct}^{cur}.$$

The k-th loop id pair $\left(q_{k}^{h},q_{k}^{c}\right)$ and the corresponding relative transformation $T_{q_{k}^{h},q_{k}^{c}}^{loop}$ are stored in an independent loop list L .

#### 3.2.2. Pose Graph Optimization and Map Reconstruction

When the loop detection is successfully triggered, we utilize the keyframe list H and loop list L to construct a pose graph optimization [30] model aimed at eliminating accumulated localization errors. The pose error terms formed by the keyframe $F^{j}$ and $F^{j+1}$ are defined as

(14) $$e_{j,j+1}=\ln\left({\bar{T}}_{j,j+1}^{-1}T_{j}^{-1}T_{j+1}\right)$$

where $T_{j}$ and $T_{j+1}$ represent the odometry poses of keyframes $F^{j}$ and $F^{j+1}$ that have not undergone loop correction. ${\bar{T}}_{j,j+1}^{-1}$ represents the relative pose transformation between two keyframes in the keyframe list H and loop list L , which is used as a measurement value in pose graph optimization. Then, the cost function corresponding to all error terms can be represented as

(15) $$\min_{T_{j}^{kf}\in H}\sum_{j=0}^{n^{kf}-1}{∥e_{j,j+1}^{T}e_{j,j+1}∥}_{2}^{2}+\sum_{q_{k}\in L}{∥e_{q_{k}^{h},q_{k}^{c}}^{T}e_{q_{k}^{h},q_{k}^{c}}∥}_{2}^{2}.$$

After loop correction, we utilize the last $n_{sl}^{near}$ keyframes in the sliding window S for map reconstruction. Based on the corrected keyframe pose and Algorithm 1, the sampling point set $\bar{\Omega }$ in each keyframe is traversed and reinserted into the H-MS map.

## 4. Results and Discussion

In this section, we validate the proposed algorithm and the integrated system. Specifically, Section 4.2 compares the insertion and deletion performance of the proposed H-MS map model with the ikd tree [15] model. Then, the modified MSBM algorithm is compared with the matching algorithm, which can also be used for the H-MS map but is based on the small-scale priority idea [20], as well as the commonly used G-ICP [21,22] and NDT [19] matching algorithms. The efficiency and accuracy of the MSBM algorithm are verified. In Section 4.3, we compare the HMS-SLAM system constructed in this article with other advanced LiDAR SLAMs (A-LOAM [31], CT-ICP [27], and VoxelMap [8]) to verify the overall mapping accuracy of the system.

### 4.1. Experimental Setup and Evaluation Metrics

The validation environment for all experiments in this article contained an AMD^®^ Ryzen 7 5800h (8 cores @ 3.2 GHz) CPU, 16 GB of RAM, and ROS Melodic. The parameter settings and evaluation metrics involved in the experiment are provided as follows.

**Parameter settings:** In the map performance experiment, the parameters of the H-MS map are set as the maximum depth d=3 and maximum scale $s_{0}=1.0$ . For the ikd tree map, the parameters include the delete parameter del=0.3 , balance parameter bal=0.6 , and box length box=0.2 .

In the matching algorithm experiment, the parameters of the MSBM algorithm are the number of neighboring voxels k=27 , the threshold for the accumulated map point number $N_{th}=10$ , the threshold for the plane landmark weight $W_{th}=0.5$ , and the matching criteria threshold $D_{th}=1.0$ m. The voxel search radius for small-scale priority matching is r=1.0 m; the parameters of $N_{th,W_{th}}$ and $D_{th}$ are as above. G-ICP and NDT follow the default settings of [32]. To mitigate frame loss due to high computational steps in G-ICP and NDT, they leverage the OpenMP [33] library for parallel acceleration processing.

In the integrated system experiment, the parameters of the H-MS map include the maximum depth d=4 and maximum scale $s_{0}=2.0$ , and the MSBM algorithm parameters remain consistent with those in the matching algorithm experiment. The parameters for keyframe detection include the time interval $\eta_{th}^{kf}=0.3$ , relative translation transformation threshold $t_{th}=1.0$ m, and relative rotation transformation threshold $R_{th}=10^{\circ }$ . The sliding window size utilized to maintain the local H-MS map is $K_{sl}=100$ . Parameters for the loop closure thread consist of the loop time interval $\eta_{th}^{loop}=20\;s$ , number of neighboring historical keyframes $n^{loop}=5$ , ICP error threshold $L_{th}=0.3$ m, and number of reconstructed keyframes $K_{sl}^{near}=7$ .

**Evaluation metric:** In the map performance experiment, we analyzed the computational efficiency of map insertion and deletion operations. All operations are timed in milliseconds. In the matching algorithm and integrated system experiments, we employed standard metrics from the SLAM field, such as ATE [34] (absolute trajectory error) and RPE [35] (relative pose error), to evaluate the proposed methods.

### 4.2. Algorithm Validation

#### 4.2.1. Map Performance Experiment

The map performance experiment is mainly divided into two parts: point insertion and voxel deletion. For the map model conducting comparative experiments, an ikd tree [15] is an improved kd tree model that can achieve incremental point insertion and deletion operations. When a new point cloud frame is inserted into the map, the ikd tree does not need to be reconstructed, making it more suitable for SLAM tasks.

**Figure 3:**
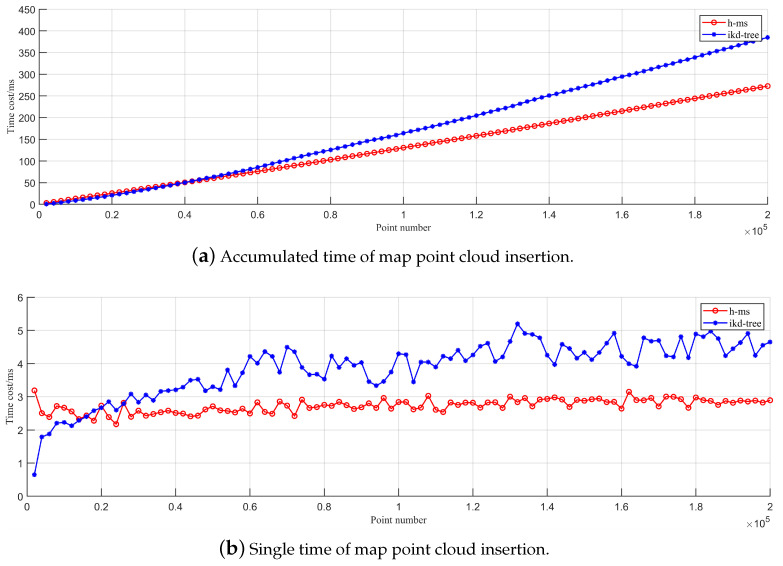
Comparison of point insertion performance between H-MS map and the ikd tree map.

In the point insertion experiment, we randomly sample 3D points within a space of 100 m × 100 m × 100 m. Subsequently, we insert 2000 points into the H-MS and ikd tree maps in each iteration, calculating the time required for the operation. Figure 3 illustrates the accumulated and single-time consumption of two models during 2 $\times \;10^{5}$ point insertions. It can be seen that the H-MS map has a better computational efficiency in the point insertion operation than the ikd tree map. This efficiency stems from the fact that a map point can access the corresponding root voxel in the H-MS map in O(1) time through the hash table. Meanwhile, the finite-time recursive insertion of a point also has the constant temporal complexity of O(1) . In contrast, the temporal complexity of point insertion in the ikd tree is $O\left(logn\right)$ .

**Figure 4:**
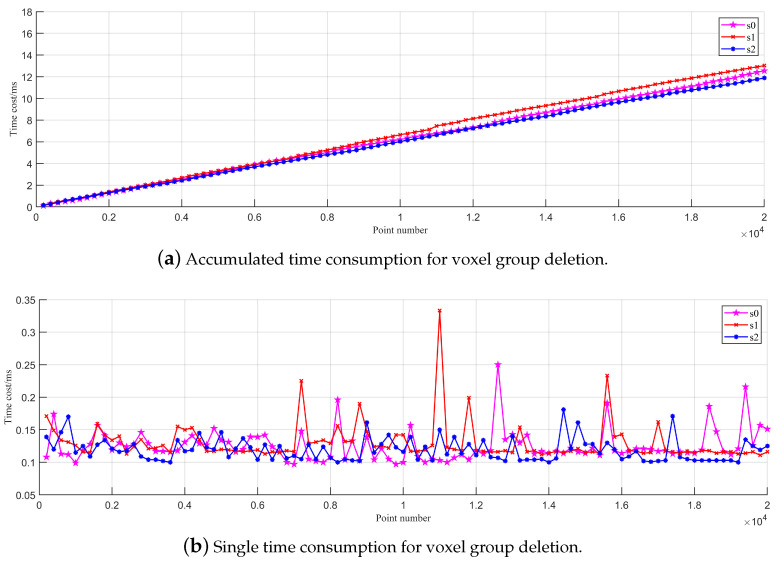
Time consumption for removing voxels from the H-MS map at different scales.

The voxel deletion experiment is subdivided into random voxel deletion and frame-based voxel deletion experiments. In the random voxel deletion experiment, we first generate an H-MS map using the method outlined in the point insertion experiment. Concurrently, the map coordinates of map voxels are stored separately by scale. Then, we record the accumulated and single computation time required to remove the map voxel set (200 voxels) at different scales. This experiment compares the H-MS map’s computation efficiency in removing voxels of different scales. Figure 4a shows that the voxel removal operation is proportional to the number of voxels being removed, validating the O(1) temporal complexity of the single voxel deletion operation. Additionally, it is worth noting that voxel deletion at the scale of $s_{1}$ always takes longer than at other scales. This observation suggests that when deleting voxels at scale $s_{1}$ , the total time required to locate the voxel from the map and remove its voxel groups is the longest.

Then, we conducted frame-based voxel deletion experiments using the outdoor KITTI odometry [35] and indoor Hilti [36] datasets. The KITTI odometry dataset offers pose ground truth and processed point cloud data without motion distortion, making it easy to construct the desired map. The Hilti dataset provides raw point cloud data with timestamps and trajectory ground truth. We applied the linear interpolation model proposed in [23] to process the raw point cloud and obtain the distortion-corrected point cloud in the LiDAR coordinate system.

Starting from frames 0, 50, 100, and so on of the sequential sequence in the dataset, we use continuous 50-frame point clouds to construct the H-MS map. Based on the voxel deletion method described in Section 3.1.3, the earliest keyframe can be removed from the sliding window. Table 1 presents the number of map voxels with observation count $l_{0}^{obs}=0$ in the earliest keyframe, along with their operation times for observation maintenance and voxel removal.

From Table 1, we found that performing frame-based voxel deletion operations on indoor Hilti datasets typically takes less time compared with outdoor autonomous driving scenes. This is because more voxels the small-scale scenes in the earliest keyframe can be observed by other keyframes in the sliding window in the small-scale scenes. These voxels do not need to be removed. For the experimental results of the Hilti-lab sequence, it is evident that both the removed number and voxel removal operation time are zero. This indicates that all observed map voxels in the earliest keyframe have a co-view relationship with other historical keyframes. Additionally, the number of voxels deleted in a single frame is always less than 500. The total cost time of the observation maintenance and voxel removal operations is less than 2 ms, which further verified the efficiency of H-MS map maintenance.

#### 4.2.2. Matching Algorithm Experiment

In this section, we conducted comparative experiments on matching algorithms using the KITTI raw [35] and ETHZ [37] datasets. The KITTI raw dataset, collected by vehicles equipped with a Velodyne HDL-64E LiDAR sensor in urban environments, provides raw point cloud data without motion distortion correction. This dataset is useful for testing the registration accuracy and computational efficiency of matching algorithms in structured scenes. The ETHZ dataset contains point cloud data collected by a Hokuyo UTM-30LX LiDAR sensor in various unstructured scenes, such as mountains and woods. This dataset is ideal for verifying the registration accuracy of matching algorithms in more challenging scenarios.

For the matching algorithms conducting comparative experiments, SSPM [20] layers voxel landmarks of different scales within a specified radius range. Then, smaller-scale voxels are prioritized for matching judgments. The algorithm can terminate in advance once the “predicate” (plane landmark weight and point number) condition is satisfied. G-ICP [21] was a modified ICP matching algorithm that considered both measurement and target covariance distribution, exhibiting good scene adaptability. Based on [21], VG-ICP [22] further achieved registration from distribution to multiple distributions using voxels. The 3D-NDT model [19] used the fixed-size grid to segment the environment and stored voxels as the normal distribution model. Table 2 and Table 3 show different matching algorithms’ registration accuracy and computational time on the real-time collected KITTI raw dataset. Table 4 shows the registration accuracy of these algorithms on the offline-collected ETHZ dataset.

As depicted in Table 2, the proposed MSBM algorithm achieved the best registration accuracy in most sequences. Furthermore, compared with the SSPM [20] algorithm, which is also applicable to the H-MS map, the MSBM algorithm yielded superior estimation results on the dataset. In applications such as ground mobile robot navigation, large-scale voxel landmarks typically enable higher precision modeling of the ground. Because the MSBM algorithm evaluates all voxels in the candidate set equally, it ensures that large-scale landmarks are not overlooked, thereby obtaining the optimal matching results.

In Table 3, G-ICP exhibits the highest operational efficiency. This is because both G-ICP and VG-ICP algorithms are implemented based on the OpenMP library. High time-consuming steps such as covariance matrix calculation and data association are accelerated through multi-threaded parallel processing. In contrast, the MSBM and SSPM algorithms only run on a single thread and require fewer computational resources. Furthermore, the computational efficiency of the MSBM algorithm is nearly twice that of the SSPM algorithm, thereby satisfying the real-time requirements of the SLAM system.

To analyze the temporal complexity of the MSBM and SSPM algorithms, we take the registration process of a 3D measurement point as an example. Assuming that the maximum depth of the H-MS map is *d* and the number of neighboring voxels for search is *k*. The MSBM algorithm uses multi-scale and finite neighboring search strategies to construct the candidate voxel set. The temporal complexity of the MSBM algorithm is O(kd) . The SSPM algorithm employs a small-scale priority search to construct the candidate voxel set, resulting in a temporal complexity of $O(8^{d})$ . Notably, as the parameter *d* increases, the computation time of the MSBM algorithm only increases linearly, whereas the computation time of the SSPM algorithm increases exponentially. This demonstrates the superior efficiency of the MSBM algorithm.

Table 4 presents the registration accuracy of different matching algorithms on the ETHZ dataset. Compared with other matching methods, the proposed MSBM algorithm achieves better registration results in most sequences. It is worth noting that during this experiment, we maintained a unique plane landmark for each voxel in the H-MS map. Although using planar parameters for environmental modeling in unstructured scenarios might seem inappropriate, the H-MS map’s ability to maintain voxels at different scales allows for effective segmentation of irregular objects like trees and grasslands. This ensures that these objects can always be segmented into a reasonably scaled voxel to find an effective local plane. Consequently, the registration accuracy of the MSBM algorithm is guaranteed in unstructured scenarios.

### 4.3. Integrated System Testing and Analysis

Compared with the KITTI raw [35] dataset, the KITTI-360 [38] dataset offers longer sequences, including point clouds larger than 1 $\times \;10^{4}$ frames, enabling the evaluation of the accuracy and stability of SLAM systems in large-scale scenarios. In this experiment, the proposed HMS-SLAM system, integrating the H-MS map and the MSBM algorithm, is compared with other advanced LiDAR SLAM frameworks using the ATE [34] and RPE [35] metrics. The ATE [34] metric evaluates the system’s absolute localization accuracy, while the RPE [35] metric reflects odometry drift. The loop closure modules were turned off during the experiment to ensure fairness in odometry verification.

For the SLAM frameworks involved in the comparison, A-LOAM [31] and CT-ICP [27] construct landmarks based on “points”, while VoxelMap [8] builds landmarks based on voxels. Specifically, A-LOAM [31] is a modified system based on [23], eliminating the use of the inertial measurement unit (IMU) sensor and relying on edge and plane feature point sets extracted from the point cloud for two-stage mapping (scan-to-scan and scan-to-map). The system organizes point cloud maps using kd trees, and each feature point in the set is matched with a landmark composed of a specified number of neighboring point set found in the kd tree. State estimation is accomplished using the Ceres 2.0.0 [39] non-linear optimizer. CT-ICP [27] maintains map points in fixed-size hash voxels and constructs landmarks using a specified number of neighboring map point sets. Additionally, the proposed continuous-time ICP algorithm considers intra-frame continuity and inter-frame non-continuity for state estimation, registering measurements to the map while removing point cloud motion distortion. Voxelmap [8] used a hash adaptive voxel map that maintained a unique plane landmark in each voxel based on the preset segmentation threshold. Moreover, Voxelmap [8] utilized an estimator based on IEKF [40] that considered both map and measurement uncertainties for robot localization.

As illustrated in Table 5, the proposed HMS-SLAM system achieved the best ATE and RPE evaluation accuracy in most sequences. For the comparison frameworks, we noticed that A-LOAM [31] and VoxelMap [8] performed worse. The A-LOAM [31] system utilized a scan-to-scan method to correct motion distortion and estimate the initial odometry pose for map refinement. However, due to the limited information in a single frame, the feature sampling set of the current frame may fail to construct a reasonable data association with the last frame. Additionally, the scan-to-scan method lacks the capability to filter data associations using the plane landmark weight, as demonstrated in [27] and our method. The hash adaptive voxel map proposed by [8] is adaptable to urban structural environments. However, the system depends on the preset threshold for the map voxel segmentation operation. This may result in the inability to create voxels at the optimal scale, thereby reducing positioning accuracy. Furthermore, we noticed that VoxelMap [8] crashed due to system memory exhaustion in sequence 02. This occurred because VoxelMap utilized all measurement information to construct a complete hash adaptive voxel map during the runtime cycle. Although the adaptive segmentation operation somewhat controls memory usage, voxel-based memory consumption is higher than that of point-based SLAM systems. Moreover, the idea of maintaining multi-scale voxels to ensure modeling accuracy for our proposed H-MS map leads to faster memory growth compared with VoxelMap. Without targeted processing, the HMS-SLAM system may struggle to achieve long-term operation. To address this issue, we introduce the frame-based voxel deletion operation in the H-MS map. Consequently, the localization and mapping tasks of the HMS-SLAM system rely only on the sliding window with a limited number of keyframes. Finally, from the results of the ATE metric, the localization accuracy of CT-ICP [27] is roughly equivalent to that of the HMS-SLAM system. However, CT-ICP [27] relied on a fixed number of neighboring point sets to construct the plane landmark, which limits the map model’s accuracy and further impacts localization results.

**Figure 5:**
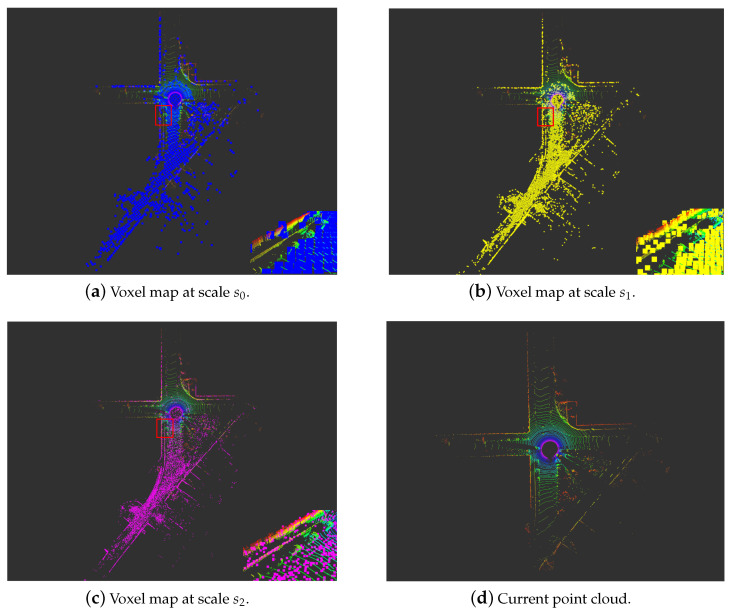
The H-MS map created by the HMS-SLAM system in sequence 10 of the KITTI-360 dataset.

Figure 5 illustrates the H-MS map constructed by the HMS-SLAM system under sequence 10 of the KITTI-360 dataset. Figure 5a–c depicts effective map voxels with a plane weight greater than 0.5 at scale $s_{0}$ , $s_{1}$ , and $s_{2}$ , respectively. The inset in the bottom-right corner provides an enlarged view of the highlighted red box. It can be seen that the effective voxels at scale $s_{0}$ mainly come from the ground. Meanwhile, most vertical walls are not regular large-scale structural planes, and only a few voxels have been successfully created. In the voxel map at scale $s_{1}$ , a greater number of effective wall landmarks can be seen. In the voxel map at scale $s_{2}$ , effective voxel landmarks are also generated for the car on the ground. By maintaining a hash multi-scale map, objects of different sizes in the environment can be accurately modeled using voxels at the corresponding scale.

## 5. Conclusions

In this article, we propose a novel hash multi-scale (H-MS) map for environmental modeling and representation. This model considers allowing a map point to simultaneously participate in creating map voxel at different scales to ensure the accuracy of map modeling. Meanwhile, by storing the root node of the multi-scale voxel group in the hash table, each group can be quickly accessed. Compared with commonly used octree and kd tree maps, the H-MS map can be used without estimating the scene size and has higher map maintenance efficiency. On the other hand, while maintaining multi-scale landmarks improves map completeness, the large number of feasible voxel landmarks also increases the difficulty of data association. To address this issue, we designed a multi-scale bidirectional matching (MSBM) algorithm. The algorithm can quickly obtain the candidate landmark set through forward–reverse–forward projection and achieve efficient data association. Then, the proposed H-MS map and MSBM algorithm are uniformly integrated into the HMS-SLAM system. Based on the publicly available KITTI dataset, we validated the maintenance efficiency of the H-MS map, the accuracy and time consumption of the MSBM algorithm, and the mapping accuracy of the HMS-SLAM system. The experimental results show that the H-MS map can complete insertion and deletion operations with O(1) temporal complexity, with higher maintenance efficiency than the ikd tree map. Compared with the small-scale priority matching algorithm, which also applies to the H-MS map, the MSBM algorithm has improved accuracy and efficiency. Moreover, the single run time of the MSBM algorithm on the CPU is around 49 ms, which meets the real-time application requirements of the SLAM system. Compared with other advanced LiDAR SLAM frameworks, the HMS-SLAM system built in this article has advantages in memory usage and mapping accuracy. In the future, we will apply a semantic segmentation module to the HMS-SLAM system to enable scene modeling that includes high-level semantic information. It is beneficial for improving the robot’s understanding and adaptability to complex environments such as high dynamics.

## Figures and Tables

**Table 1:** Time consumption and removed voxel number for frame-based voxel deletion operation under the KITTI odometry and Hilti datasets.

Sequence	Observation Maintenance [ms]	Voxel Removal [ms]	Removed Number
KITTI-00	0.874	0.602	342
KITTI-01	1.202	0.703	385
KITTI-02	1.036	0.399	204
KITTI-03	1.092	0.408	203
KITTI-04	0.946	0.445	210
KITTI-05	0.631	0.175	91
KITTI-06	1.358	0.339	192
KITTI-07	0.874	0.193	121
KITTI-08	1.187	0.479	309
KITTI-09	0.714	0.444	254
KITTI-10	0.415	0.156	73
Hilti-lab	0.127	0.000	0
Hilti-arena	0.428	0.013	6
Hilti-upper1	0.445	0.341	240
Hilti-upper3	0.205	0.076	65
Hilti-base2	0.127	0.008	2

**Table 2:** Accuracy evaluation for different matching algorithms under the KITTI raw dataset.

Absolute Motion Trajectory RMSE [m]				
Sequence	SSPM [20]	G-ICP [21]	VG-ICP [22]	MSBM (Ours)
1003-0027	15.441	6.987	10.089	**5.582**
1003-0042	**17.868**	**-**	**-**	19.816
1003-0034	43.102	**-**	**-**	**7.681**
0930-0016	**-**	**19.974**	87.848	**-**
0930-0018	11.095	**3.277**	11.502	4.623
0930-0020	19.157	3.600	2.792	**0.846**
0930-0027	8.282	**0.557**	2.034	1.301
0930-0028	**-**	**7.270**	9.293	10.975
0930-0033	4.516	7.857	46.333	**2.748**
0930-0034	3.434	3.320	23.308	**2.169**
Mean	15.312	6.605	24.150	**6.193**

Note: ‘**-**’ means the sequence was not successfully run entirely. Bold values represent the best results.

**Table 3:** Average time consumption for different matching algorithms under the KITTI raw dataset.

Average Time Consumption [ms]				
Sequence	SSPM [20]	G-ICP [21]	VG-ICP [22]	MSBM (Ours)
1003-0027	77.182	**15.929**	18.796	49.739
1003-0042	54.055	**-**	**-**	46.884
1003-0034	93.571	**-**	**-**	49.147
0930-0016	**-**	**24.766**	19.719	**-**
0930-0018	104.120	**17.503**	21.723	48.977
0930-0020	104.020	**26.295**	29.844	45.751
0930-0027	101.781	**15.694**	15.670	53.205
0930-0028	**-**	**19.896**	22.914	48.129
0930-0033	99.032	**21.287**	22.800	50.645
0930-0034	95.793	**16.216**	20.793	49.119
Mean	91.194	**19.698**	21.532	49.066

Note: ‘**-**’ means the sequence was not successfully run entirely. Bold values represent the best results.

**Table 4:** Registration evaluation for different matching algorithms under the ETHZ Dataset.

Absolute Motion Trajectory RMSE [cm]					
Sequence	SSPM [20]	G-ICP [21]	VG-ICP [22]	3D-NDT [19]	MSBM (Ours)
Apart.	2.148	**-**	**-**	14.735	**0.556**
Haupt.	1.962	**0.533**	1.293	4.892	0.621
Stairs	1.534	1.082	1.560	1.709	**0.464**
Mount.	3.078	15.407	**-**	17.727	**0.901**
Gazebo.S.	1.405	2.552	5.528	11.193	**0.741**
Gazebo.W.	1.172	4.326	3.375	6.125	**0.346**
Wood.S.	1.752	11.435	4.963	21.179	**0.500**
Wood.A.	2.384	5.343	7.691	11.951	**0.744**
Mean	1.929	5.811	4.068	11.189	**0.609**

Note: ‘**-**’ means the sequence was not successfully run entirely. Bold values represent the best results.

**Table 5:** Accuracy evaluation of different SLAM systems under the KITTI-360 dataset.

Seq.	A-LOAM [31]		CT-ICP [27]		VoxelMap [8]		HMS-SLAM (Ours)	
	ATE [m]	RPE [%]	ATE [m]	RPE [%]	ATE [m]	RPE [%]	ATE [m]	RPE [%]
00	119.753	0.631	28.847	0.810	143.471	2.845	**13.493**	**0.498**
02	227.785	0.838	77.014	0.851	**-**	**-**	**25.509**	**0.619**
03	8.680	0.252	**5.108**	0.650	102.754	**0.236**	7.006	0.556
04	110.063	0.633	-	-	85.822	1.051	**33.297**	**0.468**
05	22.331	0.775	14.039	0.864	20.152	1.467	**6.897**	**0.631**
06	135.020	**0.637**	19.795	0.825	37.853	1.193	**12.645**	0.690
07	400.692	0.616	95.663	0.808	247.970	1.785	**17.773**	**0.363**
09	236.378	0.670	31.146	0.865	144.880	0.506	**12.131**	**0.582**
10	34.223	0.450	34.974	0.861	101.053	2.837	**10.887**	**0.274**
Mean	114.361	0.611	38.323	0.816	110.494	1.49	**15.515**	**0.520**

Note: ‘**-**’ means the sequence was not successfully run entirely. Bold values represent the best results.

## Data Availability

The data presented in this work are not publicly available at this time. Raw data can be obtained upon reasonable request from the authors.
